# Genetic Gains in Grain Yield of a Maize Population Improved through Marker Assisted Recurrent Selection under Stress and Non-stress Conditions in West Africa

**DOI:** 10.3389/fpls.2017.00841

**Published:** 2017-05-22

**Authors:** Rekiya O. Abdulmalik, Abebe Menkir, Silvestro K. Meseka, Nnanna Unachukwu, Shehu G. Ado, Joseph D. Olarewaju, Daniel A. Aba, Sarah Hearne, Jose Crossa, Melaku Gedil

**Affiliations:** ^1^Maize Improvement Program, International Institute of Tropical AgricultureIbadan, Nigeria; ^2^Department of Plant Science, Institute for Agricultural Research, Ahmadu Bello UniversityZaria, Nigeria; ^3^International Maize and Wheat Improvement CenterMexico City, Mexico

**Keywords:** maize, bi-parental cross, MARS, genetic gain, allele frequency

## Abstract

Marker-assisted recurrent selection (MARS) is a breeding method used to accumulate favorable alleles that for example confer tolerance to drought in inbred lines from several genomic regions within a single population. A bi-parental cross formed from two parents that combine resistance to *Striga hermonthica* with drought tolerance, which was improved through MARS, was used to assess changes in the frequency of favorable alleles and its impact on inbred line improvement. A total of 200 testcrosses of randomly selected S_1_ lines derived from the original (C_0_) and advanced selection cycles of this bi-parental population, were evaluated under drought stress (DS) and well-watered (WW) conditions at Ikenne and under artificial *Striga* infestation at Abuja and Mokwa in Nigeria in 2014 and 2015. Also, 60 randomly selected S_1_ lines each derived from the four cycles (C_0_, C_1_, C_2_, C_3_) were genotyped with 233 SNP markers using KASP assay. The results showed that the frequency of favorable alleles increased with MARS in the bi-parental population with none of the markers showing fixation. The gain in grain yield was not significant under DS condition due to the combined effect of DS and armyworm infestation in 2015. Because the parents used for developing the bi-parental cross combined tolerance to drought with resistance to *Striga*, improvement in grain yield under DS did not result in undesirable changes in resistance to the parasite in the bi-parental maize population improved through MARS. MARS increased the mean number of combinations of favorable alleles in S_1_ lines from 114 in C_0_ to 124 in C_3_. The level of heterozygosity decreased by 15%, while homozygosity increased by 13% due to the loss of some genotypes in the population. This study demonstrated the effectiveness of MARS in increasing the frequency of favorable alleles for tolerance to drought without disrupting the level of resistance to *Striga* in a bi-parental population targeted as a source of improved maize inbred lines.

## Introduction

Maize (*Zea mays* L.) is an important food security and income-generating crop for millions of people in West and Central Africa (Adetimirin et al., 2008). Its production is limited by several biotic and abiotic factors including *Striga hermonthica* (Del) Benth, drought, declining soil fertility and susceptibility to pests and diseases (Odendo et al., 2001). Globally, about 160 million hectares of maize is grown under rain-fed conditions and annual yield losses attributed to drought are estimated at about 25% (Edmeades, 2008). The losses are expected to be greater in tropical countries that rely on unpredictable and erratic rainfall (Mhike et al., 2012). The gap between potential yield and yield under drought stress (DS) is often large, but 20–25% of this gap could be eliminated by genetic improvement in drought tolerance and a further 20–25% by application of water-conserving agronomic practices (Edmeades, 2013). The remaining 50–60% can only be met by irrigation, when available and affordable (Edmeades et al., 2006). Drought alone causes an average yield loss of about 17–60% (Edmeades et al., 1999), while *Striga* causes an estimated yield loss of about 10–100% under severe infestation (Lagoke et al., 1991; Odhiambo and Woomer, 2005). Armyworm (*Spodoptera* spp.) infestation occurs in maize from plant emergence to tasseling and silking. Losses due to the fall armyworm attack can reduce grain yield up to 34% (Cruz et al., 1999; Lima et al., 2010). Marker-assisted recurrent selection (MARS) in combination with high-throughput and precise phenotyping and year round nurseries can significantly accelerate the development of climate resilient maize germplasm and have been used to improve tolerance to drought (Xu et al., 2012; Prasanna et al., 2013).

Marker-assisted recurrent selection for quantitative traits has relied on identifying markers linked to quantitative trait loci (QTL). It involves improvement of an F_2_ population by one cycle of selection based on phenotypic data and marker scores followed by two to three cycles of marker based scores only (Johnson, 2001, 2004). Improving bi-parental maize populations for tolerance to stress through MARS is of major importance because it harnesses several QTLs carrying the most desirable combinations of favorable alleles using only significant markers to predict performance of the population (Meuwissen et al., 2001; Bohra, 2013). This procedure has been effective and superior to phenotypic selection (PS) by accumulating favorable alleles for multiple trait improvement in maize and other crops (Edwards and Johnson, 1994; Van Berloo and Stam, 1998, 2001; Charmet et al., 1999; Johnson, 2001, 2004; Yousef and Juvik, 2001; Eathington et al., 2007; Crossa et al., 2013, Massman et al., 2013).

Assessment of the changes in the frequency of favorable alleles within a bi-parental population improved by MARS would provide information on specific genomic regions that have responded to selection (Frascaroli and Landi, 1998). In addition, assessment of genetic gains in breeding program provides an opportunity to critically analyze efficiency and plan new actions and strategies. Realized progress with any breeding scheme, however, depends largely upon the ability of the breeders to identify superior genotypes and the precision of experimentation (Khalil et al., 2010). Xu et al. (2012) proposed MARS as an effective tool to breed for complex traits because it enables the harnessing of those genes or QTL exhibiting minor effects on the phenotype. Edwards and Johnson (1994) studied the changes in frequency of favorable alleles in sweet corn F_2_ MARS population and observed an increase in the frequency of the favorable alleles from 0.50 to ≥0.80 at 11 out of 35 markers used and one marker locus in the population became fixed for the favorable allele. Bernardo and Mayor (2009) observed an increase in the frequency of favorable alleles for grain yield from C_0_ (0.50) to C_2_ (0.65) in a double haploid (DH) population improved by MARS. Recent studies (Semagn et al., 2015; Beyene et al., 2016a) have highlighted improved genetic gains in grain yield of tropical maize populations using MARS under DS. Semagn et al. (2015) reported an average gain of 184 kg ha^-1^ cycle^-1^ under WW and 45 kg ha^-1^ under DS conditions in bi-parental maize populations, whereas Beyene et al. (2016a) reported an average gain of 105 kg ha^-1^ year^-1^ under well-watered (WW) and 51 kg ha^-1^ year^-1^ under DS.

Maize breeders at the International Institute of Tropical Agriculture (IITA) developed a bi-parental maize population from elite inbred parents with combined tolerance to drought and resistance to *S. hermonthica*. The focus of the breeding activities was to improve the population for tolerance to drought in order to extract superior inbred lines with enhanced recombination of favorable alleles originating from both parents. The bi-parental population was improved through three cycles of marker assisted recurrent selection under DS condition. However, studies have not been conducted to assess genetic gains in tolerance to drought and changes in the frequency of favorable alleles that accrued under drought stressed conditions in this population. Also, assessment of the performance of progenies derived from improved cycles of this population under *S. hermonthica* infestation as a non-target environment is important to determine the effect of parental selection on grain yield and resistance to the parasite. This study was therefore, conducted to assess (i) genetic gains in grain yield under DS and WW conditions, (ii) the potential impact of parental selection on non-target traits under *Striga* infested condition in a bi-parental population improved with MARS, and (iii) the associated changes in the frequency and number of favorable alleles using SNP markers.

## Materials and Methods

### Development of MARS Population, Phenotyping and Genotyping of F_2:3_

The population targeted for MARS in the study was derived from a cross between two elite *Striga* resistant inbred lines (Acr.Syn-W-S_2_-173-B^∗^4) and (TZLComp.1C_4_-S_1_-37-5-B^∗^3), that are also tolerant to drought and resistant to the major lowland foliar diseases. The F_1_ was selfed to generate F_2_ bulk seeds, which were grown in 50 rows of 5 m length spaced 0.75 m apart to generate 300 F_2:3_ lines (F_2_ derived populations in F_3_). A total of 250 F_2:3_ lines from this population were planted each in a row and crossed to an inbred tester from an opposite heterotic group. The testcrosses were evaluated under DS and WW conditions at Ikenne during the dry season (Supplementary Figure S1).

Marker effects of the genotyped 250 F_2:3_ lines were calculated using the best linear unbiased prediction (BLUP) model (Meuwissen et al., 2001), which permitted predicting genomic estimated breeding value (GEBV) (Henderson, 1975; Gianola and Fernando, 1986). The GEBV was calculated per marker across all the lines derived from C_0_ using the BLUP values. Each line was scored 0 or 1 based on the presence or absence of parental allele. The BLUP value per marker was multiplied by the marker score of each line and the resultant values per line were the GEBVs. Significant markers on each chromosome were identified by backward elimination. A relaxed significance level (*P* = 0.10), which has been found desirable to maximize the response to MARS (Hospital et al., 1997; Johnson, 2001) was used. Selection at C_1_ was based on marker used to calculate GEBV which is the sum of all marker effects included in the model for an individual. The selected C_0_ lines were ranked according to their GEBV and 10% of the S_1_ lines with the highest GEBV were planted ear-to-row and inter-crossed. Bulk pollen collected from 10 plants in each line was used for inter-crossing with other lines. Four ears were harvested in each row and shelled to obtain more than 100 seeds per ear. Equal amounts of seed were taken at random from each ear to form a bulk of the new cycle (C_1_) for planting. Leaf samples were collected from each of the plants for genotyping at LGC Genomics using the full complement of SNPs originally used for genotyping the C_0_ populations. The top 10% of the C_1_ individuals were selected based on GEBV and intermated to form C_2_ as described above and repeated to form C_3_. All recombination were conducted at IITA, Ibadan in Nigeria.

### Formation of Testcrosses for Phenotypic Evaluation

In 2013, the original (C_0_) and advanced selection cycles (C_1_, C_2_, and C_3_) of the MARS population were planted at Ibadan each in 60 rows of 5 m length spaced 0.75 m apart. Several plants were self-pollinated and 120 to 150 S_1_ lines were harvested from each selection cycle and retained. Amongst these, 60 S_1_ lines were randomly selected and were planted along with parental lines of the bi-parental cross and an inbred tester (TZISTR1138) at Ibadan in 2013 to generate testcrosses. The S_1_ lines were used as female parents, whereas the inbred tester was used as the male parent. Bulk pollen collected from the male parent was used to pollinate the emerged silks of several plants in each S_1_ lines. The ears from each testcross were harvested and dried to 15% moisture content and shelled.

### Selection of Markers

A final set of 275 SNPs were selected after rigorous screening of 1250 KASP assays developed by LGC Genomics (United Kingdom) by converting 1536 Illumina Golden Gate Array (Semagn et al., 2013). Two separate bulks of leaf from eight plants of each parent and one bulk of F_1_ was genotyped with all 1247 SNP markers at LGC Genomics (formerly KBiosciences, United Kingdom). Of the 1247 markers run by LGC Genomics markers over a 1000 SNPs provided successful calling in one or both bulks of each parent. However, SNPs that were not uniform between the two bulks (one bulk homozygous and the other heterozygous; one has allele call and the other has no allele call) of the same parent or that gave no call in one or the other parent were eliminated. Likewise, SNPs that were heterozygous in either parent or homozygous in the F_1_s were also excluded. Finally, 233 markers that are uniform and homozygous in the parents as well as polymorphic between the parents were used for genotypic selection in the MARS population (Supplementary Data 1).

### DNA Extraction and Genotyping

Sixty randomly selected maize sample from each MARS cycles were used for DNA extraction. Leaf samples from each of the randomly selected 60 S_1_ lines planted to generate testcrosses and the two parents were collected 2 weeks after planting and transported to IITA Biosciences laboratory in Ibadan, Nigeria for DNA extraction. The samples were lyophilized to dry for about 3 days, then two tiny steel grinding balls (2.4 mm) were inserted into each extraction tubes. About 15–20 small leaf disk of each sample was punctured into extraction tubes, covered up and grounded into fine powder using the Geno Grinder–2000. Genomic DNA was extracted using a CTAB extraction protocol of Azmach et al. (2013). The clean pellets were dried by leaving the tubes open for at least an hour to get rid of all drops of ethanol. The dried DNA (pellet) in each tube was dissolved in 100 μl solution of Rnase–DNase free water to get rid of all traces of RNA in the pellet. The purified genomic DNA was quantified in ng/ųl on a Nanodrop spectrophotometer and ran on 1% agarose gel to double check the DNA quality. Genomic DNA samples were lyophilized to dry powder and sent to LGC genomics (United Kingdom) for single nucleotide polymorphism (SNP) genotyping on Kbiosciences’ KASP assay platform (KBioscence-LGC Genomics)^1^. The SNP data obtained from this assay was used to assign genotype score to the population.

### Phenotypic Evaluation of Testcrosses

An experiment consisting of 50 randomly selected testcrosses of S_1_ lines from each of the four cycles of selection, along with testcrosses of each of the parental line of the bi-parental cross to the same tester (P_1_ × T, P_2_ × T), a cross of the two parental lines (P_1_ × P_2_) and standard hybrid checks 9022-13 and 8338-1 were evaluated under DS and WW conditions at Ikenne (6°53′ N, 30°42′ E, 60 m asl, 1200 mm annual rainfall) during the 2014 and 2015 dry seasons. The testcrosses were arranged in a 41 × 5 alpha lattice design with two replications and were planted in single rows of 5 m long with 0.75 m space between rows and 0.25 m spacing between plants within a row (Abdulmalik et al., 2016). In the DS trial, DS was imposed by withdrawing irrigation water from 5 weeks after planting through harvest, whereas the WW trial received irrigation until physiological maturity. NPK 15:15:15 fertilizer was applied at the rate of 60 kg N ha^-1^, 60 kg P ha^-1^, and 60 kg K ha^-1^, at planting and an additional 60 kg N ha^-1^ was applied 4 weeks later. In each trial, gramoxone and atrazine were applied as pre-emergence herbicides at 5.0 l ha^-1^. Subsequently, manual weeding was done to keep the experiments weed-free.

The testcrosses were also evaluated under *Striga* infestation at Abuja (9°16′ N, 7°20′ E, 300 m asl, 1500 mm annual rainfall) and Mokwa (9°18′ N, 5°04′ E, 457 m asl, 1100 mm annual rainfall) for 2 years. The *S. hermonthica* seeds for infestation were mixed with fine sand in the ratio of 1:99 by weight and about 5,000 germinable *Striga* seeds were placed in each planting hills as described by Kim (1991). NPK 15:15:15 fertilizer was applied at the rate of 30 kg N ha^-1^ at planting, and an additional 30 kg N ha^-1^ was applied 4 weeks after planting. The N rate, which was only half the recommended rate for maize in the savannas of Nigeria, was used to ensure optimal development of *S. hermonthica* that allowed differentiation among testcrosses for *Striga* damage rating (SDR) and ensured a minimum of 50% yield reduction under infestation. Weeds other than *Striga* were removed by hand throughout the planting season.

### Data Collection

Data was collected for grain yield, days to silking, anthesis-silking interval (ASI), plant height, ear aspect (EASP), plant aspect (PASP), and leaf senescence (SEN), SDR at 8 and 10 weeks after planting and number of emerged *Striga* plants (ESP). Days to silking was recorded as the number of days from planting to when 50% of the plants showed emerged silks. ASI was computed as the interval in days between silking and anthesis. Plant height was measured as the distance from the base of the plant to the height of the first tassel branch. EASP was also visually rated on a scale of 1 to 5, where 1 = clean, uniform, large, and well-filled ears and 5 = rotten, variable, small, and partially filled ears. PASP was rated on a scale of 1 to 5, where 1 = excellent overall phenotypic appeal and 5 = poor overall phenotypic appeal. SEN was scored on a scale of 1 to 9, where 1 = almost all leaves green and 9 = virtually all leaves dead. SDR was recorded on a scale of 1–9 (Kim, 1991) at 8 and 10 weeks after planting, where 1 = no damage (highly resistant), 9 = severe damage (highly susceptible). Number of ESP was counted at 8 and 10 weeks after planting. All ears harvested from each plot were shelled to determine percentage moisture and grain yield adjusted to 15% moisture was computed from the shelled grain weight.

Percentage yield loss=yield of (WW-DS)/WW*100

### Statistical Analysis

Analysis of variance (ANOVA) combined over years for all traits measured was conducted with PROC GLM in SAS using a RANDOM statement with the TEST option (SAS Institute, 2009). Independent ANOVA were conducted for data collected under DS, WW, and *Striga* infested conditions. Years, environments, replications, and incomplete blocks were considered as random effects whereas selection cycles and testcross within cycles were considered as a fixed effect. The significance of mean squares for the main and interaction effects were tested using the appropriate mean squares, obtained from the RANDOM option in SAS (SAS Institute, 2009).

For each trait, cycle means across environment were regressed as dependent variables on cycle numbers (0–3) as independent variables. The coefficient of linear regression (*b*-value) provided an estimate of the gain per cycle, which was divided by the intercept and multiplied by 100 to obtain the percent response per cycle.

Favorable alleles are alleles that have a positive effect on the trait under selection for higher values and negative effect on the trait under selection for lower values. The favorable allele of each marker was determined using the coded marker scores -1 or 1 that was used to represent each parent. As the target trait was grain yield and alleles were expected to have a positive effect on it, the parent with the positive marker score was chosen as the favorable parent. The mean change in the frequency of favorable alleles was calculated for each cycle for all markers using the Power Marker (v3.25) software. The mean, minimum, maximum, skewness, kurtosis, and standard error of the frequency of favorable alleles were calculated using the PROC UNIVARIATE procedure in SAS version 9.3 (SAS Institute, 2009). The minor allele frequency, inbreeding coefficient, heterozygosity, homozygosity, number of effective alleles, genotype lost, and genotype gained were calculated using the Power Marker (v3.25) software.

## Results

### Performance of Testcrosses of S_1_ Lines under Drought Stress and Well-Watered Conditions

In the combined ANOVA, year was a significant source of variation for all measured traits except ASI, plant height, PASP, SEN under DS and ASI, and EASP under WW conditions (**Table 1**). The variation of testcrosses within cycle was highly significant for days to silking and SEN under DS and days to silking, ASI and plant height under WW conditions. Year × testcross interaction was significant only for days to silking under both DS and WW conditions (**Table 1**).

**Table 1:** Combined mean squares from analysis of variance for grain yield and other agronomic traits of a population improved with MARS under drought stress and well-watered conditions at Ikenne in 2014 and 2015.

Source	df	Grain yield (kg ha^-1^)	Days to silking	ASI	Plant height (cm)	Ear aspect (1–5)	Plant aspect (1–5)	Leaf senescence (1–9)
**Drought stress**								
Year	1	503468519^∗∗∗^	3738.9^∗∗^	96.2	71103	22.7^∗^	7.1	23.2
Rep (year)	2	1095999	46.5^∗∗∗^	29.6^∗^	27823^∗∗∗^	1.2^∗^	1.3	2.6
Block (rep^∗^year)	160	347250^∗∗∗^	4.9^∗∗∗^	2.0^∗∗^	769^∗∗∗^	0.3^∗∗∗^	0.4^∗∗∗^	1.1^∗∗∗^
Cycle	3	609210	1.2	0.6	512	0.3	1.4^∗∗∗^	1.8
Testcross (cycle)	196	173602	6.1^∗∗∗^	1.6	194	0.1	0.2	0.8^∗∗∗^
Year^∗^cycle	3	126009	1.2	0.6	277	0.1	0.2	1.4
Year^∗^testcross (cycle)	196	164532	2.79^∗^	1.4	160	0.1	0.1	0.5
Error	238	154694	2.2	1.5	161	0.1	0.2	0.6
**Well-watered**								
Year	1	270273062^∗∗∗^	1055.7^∗^	0.3	61347.2^∗∗∗^	3.0	55.2^∗∗∗^	
Rep (year)	2	543111	4.9	0.8	203.8	0.2	0.0	
Block (rep^∗^year)	160	1137466^∗∗∗^	3.4^∗∗∗^	0.4	348.2^∗∗∗^	0.2^∗∗∗^	0.2^∗∗∗^	
Cycle	3	181768	2.4	0.4	201.7	0.1	0.1	
Testcross (cycle)	196	842886	5.4^∗∗∗^	0.6^∗∗∗^	192.8^∗∗∗^	0.1	0.2	
Year^∗^cycle	3	308202	0.9	0.6	109.6	0.2	0.1	
Year^∗^testcross (cycle)	196	808615	2.3^∗^	0.4	104.2	0.1	0.2	
Error	238	697218	1.8	0.4	120.8	0.1	0.2	


*Significant at ^∗^*P* ≤ 0.05, ^∗∗^*P* ≤ 0.01, and ^∗∗∗^*P* ≤ 0.001 levels, respectively.*

Significant mean squares were recorded among selection cycles for grain yield, days to silking, and plant height under *Striga* infested condition (Table not included). Testcrosses within cycles were significant for all traits except for EASP and SDR at 8 weeks after planting. Environment × testcross interaction was significant for days to silking and SDR at 8 weeks after planting, while environment × cycle interaction was not significant for the remaining other measured traits.

### Genetic Gains of a Bi-parental Population Improved with MARS

The testcrosses of MARS population sustained a 87% yield loss in 2015 due to severe damage by fall armyworm and an average of 73% in both years. The standard hybrid check (9022-13) also had the largest yield loss of 88% under DS, the highest days to silking, poorest PASP and shortest plants (**Table 2**). The highest grain yield was recorded at C_3_, which was significantly higher than the two standard check hybrids (9022-13 and 8338-1). The C_3_ yielded 13% more than C_0_, 10% above P_1_xT and 44% more than 8338-1 (**Table 2**). The C_3_ out yielded the base population by 163 kg ha^-1^ in the DS condition and also sustained a yield loss of 71% between the WW and DS conditions (**Table 2**). In general, mean ASI was 3 days under DS and 2 days under WW condition. Under *Striga* infestation, traits measured did not change with selection in the population through MARS. However, the testcrosses of S_1_ lines derived from the various cycles produced more yield, had taller plants, supported fewer *S. hermonthica* plants, had improved *Striga* ratings and better EASP scores compared to the hybrid checks (**Table 3**).

**Table 2:** Means and genetic gains for grain yield and other agronomic traits of a population improved with MARS under drought stress and well-watered condition at Ikenne in 2014 and 2015.

	Grain yield (kg ha^-1^)		Days to silking (days)		ASI		Plant height (cm)		Ear aspect (1–5)		Plant aspect (1–5)		Leaf Senescence (1–9)	
							
Genotypes	DS	WW	DS	WW	DS	WW	DS	WW	DS	WW	DS	WW	DS
C_0_	1280	5013	58	58	3	2	151	201	3.4	2.8	3.0	2.5	6.3
C_1_	1387	5111	58	58	4	2	156	204	3.3	2.7	2.8	2.4	6.1
C_2_	1347	5045	58	58	3	2	154	204	3.3	2.7	2.8	2.4	6.3
C_3_	1443	5055	58	58	3	2	155	204	3.3	2.7	2.8	2.4	6.3
P_2_ × Tester	1156	5334	58	60	3	2	164	203	3.1	2.5	2.9	2.4	6.8
P_1_ × Tester	1317	6402	58	58	3	2	155	212	3.4	2.5	3.1	2.1	7.0
P_1_ × P_2_	1228	5752	59	59	4	2	165	221	3.4	2.3	2.8	2.1	6.3
9022-13 (sus)	323	2587	64	62	3	1	128	181	4.0	3.3	3.6	2.8	5.3
8338-1(sus)	1003	3174	63	62	2	2	163	197	3.6	3.5	2.9	2.6	5.3
LSD_(0.05)_	ns	ns	ns	ns	ns	ns	ns	ns	ns	ns	0.01	ns	ns
CV (%)	29	17	3	3	35	33	8	6	11	13	14	17	12
Gain cycle^-1^	44.9	6.0	-0.02	0.11	-0.1	0.03	0.9	0.9	-0.03	-0.03	-0.06	-0.03	0.02
% Resp cycle^-1^	3.6	0.1	-0.03	0.2	-2.9	1.5	0.6	0.4	-0.9	-1.1	-2.0	-1.2	0.3
*R*^2^	0.71	0.04	0.07	0.69	0.07	0.60	0.36	0.60	0.60	0.60	0.60	0.60	0.07


*Resp, Response; Sus, Susceptible.*

**Table 3:** Means and genetic gains for grain yield and other agronomic traits of testcrosses of a population improved with MARS under *Striga* infested condition at Abuja and Mokwa in 2014 and 2015.

Genotypes	Grain yield (kg ha^-1^)	Days to silking	Plant height (cm)	*Striga* damage rating 8 weeks (1–9)	*Striga* damage rating 10 weeks (1–9)	Emerged *Striga* count 8 weeks (number)	Emerged *Striga* count 10 weeks (number)	Ear aspect (1–5)
C_0_	3839	61	151	2.5	4.0	2.3	2.7	3.1
C_1_	4055	61	157	2.4	3.9	2.2	2.6	3.0
C_2_	4024	61	155	2.5	3.9	2.3	2.7	3.0
C_3_	3824	61	156	2.4	4.0	2.2	2.6	3.0
P_2_ × Tester	3220	62	155	2.6	4.1	2.1	2.8	3.1
P_1_ × Tester	3690	61	146	2.3	3.8	2.2	2.5	3.0
P_1_ × P_2_	4359	62	162	2.3	3.9	2.3	2.7	2.7
9022-13 (tolerant)	1371	66	144	3.5	6.5	2.6	3.1	3.6
8338-1 (susceptible)	406	57	126	5.1	6.7	2.7	2.9	4.6
LSD_(0.05)_	108	0.2	1.5	ns	ns	ns	ns	ns
CV (%)	20	4	7	21	20	31	23	12
Average gain cycle^-1^	-7.6	0.07	1.3	-0.02	0.03	-0.02	-0.02	-0.03
% response cycle^-1^	-0.2	0.1	0.9	-0.8	0.6	-0.9	-0.7	-1.0
R^2^	0.01	0.52	0.41	0.20	0.12	0.20	0.20	0.60


The response to selection per cycle was -2% for PASP under DS condition (**Table 2**).

**FIGURE 1:**
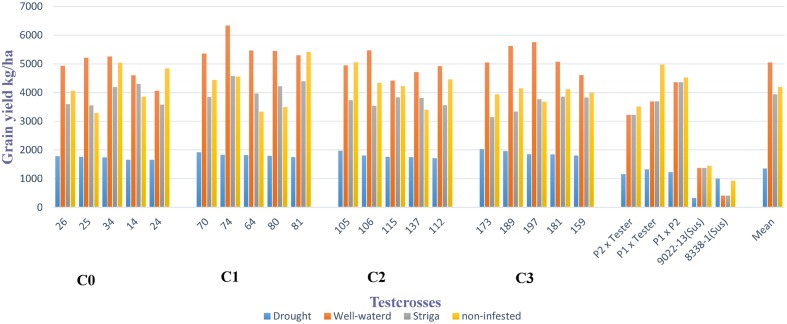
**Mean grain yield of the best five testcrosses selected from each cycle of MARS population under drought, well-watered, *Striga* infested and non-infested conditions at Ikenne, Abuja, and Mokwa in 2014 and 2015**.

Grain yield of the best testcrosses in each selected cycle of the MARS population under DS with their corresponding performance under WW condition, *Striga* infested and non-infested conditions are presented in **Figure 1**. The best testcrosses differed significantly from the parents, P_1_ × P_2_ and hybrid checks under DS condition. The performance of the testcrosses ranged from 35 to 84% above P_1_ × P_2_ and hybrid checks under DS condition. However, the performance of the testcrosses above P_1_ × P_2_ and hybrid checks were not consistent under WW condition, *Striga* infested and non-infested conditions.

### Changes in Frequency of Favorable SNP Marker Alleles

**FIGURE 2:**
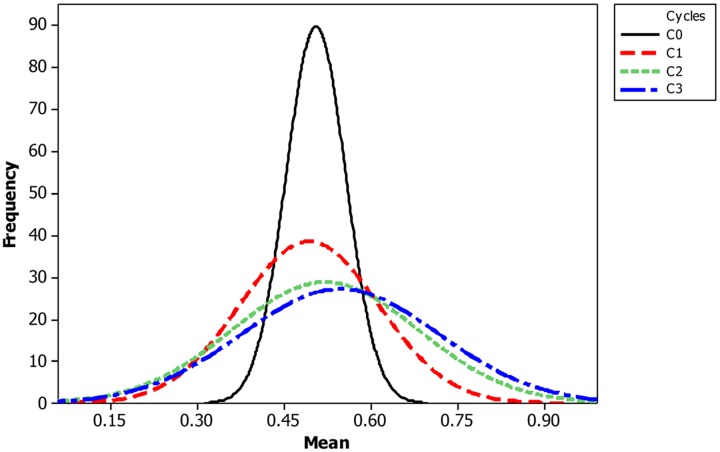
**Changes in frequency of favorable alleles from C0 to C3 in the bi-parental population**.

The mean frequency of the favorable marker alleles for grain yield increased from C_0_ to C_3_ by 9% (**Table 4**). In C_1_, the frequencies of the favorable alleles fell below the expected frequency (0.50) and picked up again in the advanced cycles (**Figure 2** and **Table 4**). None of the markers were fixed for the favorable allele in the different cycles of MARS (**Table 4**). The mean combination of favorable alleles present in each S_1_ lines increased significantly by 8% from C_0_ to C_3_ and the mean of the best 10 S_1_ lines cycle^-1^ increased from C_0_ to C_3_ by 7% (**Table 5**). The change in minor alleles decreased by 22% with advance in selection from C_0_ to C_3_, but none of the marker loci got fixed (**Table 6**). The level of heterozygosity among the cycles decreased by 15% and the number of effective alleles decreased by 9% from C_0_ to C_3_. The inbreeding coefficient increased by 57% and homozygosity by 13% from C_0_ to C_3_. About 5% of the total markers used were lost during selection at C_2_ and C_3_ (**Table 7**). Twelve genotypes were lost at C_2_ and C_3_ and only two of the markers (bt2_7 and PZA02148_1) were common in the two cycles (**Table 7**).

**Table 4:** Means, maximum, and minimum frequencies of favorable marker alleles for yield.

Cycles	C_0_	C_1_	C_2_	C_3_
Minimum	0.33	0.23	0.19	0.08
Maximum	0.76	0.80	0.95	0.91
Skewness	0.86	0.21	0.21	0.16
Kurtosis	3.49	-0.63	-0.67	-0.35
Mean	0.50 ± 0.01	0.49 ± 0.01	0.52 ± 0.01	0.55 ± 0.01


**Table 5:** Means, maximum, and minimum number of combinations of favorable marker alleles present in all and the best 10 S_1_ lines of each MARS cycle.

Entries	Minimum favorable alleles in S_1_	Maximum favorable alleles in S_1_	Mean of favorable alleles in S_1_
C_0_S_1_ lines	54	132	114 ± 1.6
Best 10 C_0_S_1_ lines	125	132	128 ± 1.0
C_1_S_1_ lines	8	132	111 ± 2.1
Best 10 C_1_S_1_ lines	124	132	128 ± 1.0
C_2_S_1_ lines	86	138	117 ± 1.3
Best 10 C_2_S_1_ lines	127	138	130 ± 1.3
C_3_S_1_ lines	87	145	124 ± 1.5
Best 10 C_3_S_1_ lines	134	145	138 ± 1.0


**Table 6:** Allelic pattern of MARS population genotyped using single nucleotide polymorphism (SNP) markers.

Cycles	Minor allele frequency	Inbreeding coefficient	Heterozygosity	Homozygosity	Number of effective alleles
C_0_	0.46	0.03	0.48	0.52	1.98
C_1_	0.40	0.04	0.45	0.55	1.90
C_2_	0.37	0.06	0.42	0.58	1.83
C_3_	0.36	0.07	0.41	0.60	1.81


**Table 7:** The loss of genotypes in SNP markers across MARS cycles.

Markers	C_0_	C_1_	C_2_	C_3_
bt2_4	++	++	++	A/A
bt2_7	++	++	G/G	G/G
PHM1190_3	++	++	++	A/A
PHM3334_4	++	++	++	G/G
PHM3334_6	++	++	++	G/G
PHM3587_6	++	++	++	G/G
PZA00311_5	++	++	++	A/A
PZA00613_22	++	++	++	C/C
PZA02148_1	++	++	G/G	G/G
PZA02260_2	++	++	++	C/C
PZA03270_2	++	++	++	A/A
PZA03597_1	++	++	++	A/A


*++, Genotype is present in the cycle; A/A, G/G, C/C, missing genotypes.*

## Discussion

Developing hybrids that are able to withstand DS throughout the growing season with no yield penalty under optimum conditions have become important, since drought incidence and severity vary considerably among years and within fields (Beyene et al., 2016a). The year × cycle interaction was not significant for any of the measured traits under both DS and WW conditions, suggesting that cycles showed consistent performance despite the stress conditions, across the 2 years. This was consistent with the result of other authors (Menkir and Kling, 2007; Derera et al., 2008; Menkir et al., 2010; Adebayo and Menkir, 2014) but was in disagreement with Menkir et al. (2012). Consequently, genotypes with consistently better drought tolerance and high yield potential can be selected under both conditions.

The absence of significance among cycles and the high level of yield reduction observed in this study under DS condition, resulted from the combined effect of severe DS and armyworm (*Spodoptera* spp.) infestation that occurred in 2015. DS can cause detrimental effects to plant pathogen resistance (Atkinson and Urwin, 2012). Exposure of plants to a pest or pathogen increases the effects of an abiotic stress such as water deficit (Cockfield and Potter, 1986; Audebert et al., 2000). For these reasons, the observed yield reduction under DS in the present study was more than those reported by other authors (NeSmith and Ritchie, 1992; Banziger et al., 2000; Menkir and Akintunde, 2001; Campos et al., 2006; Derera et al., 2008). Though, an increase in mean performance for grain yield, plant height and a decrease in PASP and SEN ware observed in the bi-parental population, the observed shift with selection moved towards the positive direction. This was consistent with the result of Beyene et al. (2015), who also reported an increase in grain yield and plant height. The ASI increased up to 4 days under DS condition from an average of 2 days under WW condition. These results are in agreement with the findings of earlier studies (Edmeades et al., 1995; Menkir and Akintunde, 2001; Kamara et al., 2003), who reported that ASI is a useful adaptive trait for selecting maize for drought-tolerance. Days to silking did not differ for all cycles, indicating that the lines were at similar phenological stage (Magorokosho et al., 2003). MARS cycles for plant height and SEN were significantly (*P* < 0.05) different from each other and from the hybrid checks. Beyene et al. (2016a) also found significant difference among the populations for plant height under DS.

The non-significant gain for grain yield observed in this study under DS, was not consistent with the findings of previous empirical and simulation studies (Bernardo and Yu, 2007; Massman et al., 2013; Beyene et al., 2016a). The lack of response to selection under WW condition for grain yield and other traits was consistent with the findings of previous studies (Bänziger et al., 1999; Magorokosho et al., 2003). Beyene et al. (2015) and Semagn et al. (2015) conducted MARS on different bi-parental maize populations under DS and WW environments and observed that each population deferred in their response to MARS.

The focus of MARS on selection for high yield under managed DS alone did not result in an overall improvement in genetic gain for grain yield and resistance to the parasite. Nonetheless, the selection for tolerance to drought did not have any negative effect on the performance of testcrosses of the different selection cycles when compared to the parents and hybrid checks. As selection for drought tolerance resulted in a negative response to selection for grain yield under *Striga* infestation (non-target traits), it is important to choose parents with tolerance to multiple stress and conduct selection under the different stress conditions to attain the desirable improvement in performance of the progenies derived from bi-parental cross. This was in agreement with the findings of Casler et al. (2003), who reported little improvement on the non-target trait from the simultaneous improvement of forage yield and seed yield of orchard grass bred for an increase in forage yield. Contrary to these findings, Lafitte and Edmeades (1995) and Edmeades et al. (2006), reported significant improvements in the non-target trait in a research selected for tolerance to drought. These could probably be due to genetic and physiological mechanism(s) between the parental traits. In rare situations, selection for a non-target trait proves more effective than the target trait and when it occurs, it may simply be that the parental alleles under selection are more or less the same in both environments or because the environments do not differ sufficiently (Witcombe et al., 2008).

Testcrosses 14, 34, 64, 74, 80, and 81 showed consistent performance above the mean under both DS and *Striga* infested conditions. As shown in **Figure 1**, the best testcrosses derived from the bi-parental cross produced higher mean grain yields than the parents, P_1_ × P_2_ and hybrid checks under DS condition. This result is similar to the findings of Beyene et al. (2016b), who reported higher performance in grain yield of MARS derived lines compared to parents and hybrid checks under DS condition.

The increase in mean frequency of the favorable marker alleles for grain yield from C_0_ to C_3_ indicated that MARS rapidly accumulates favorable alleles linked to the desired QTLs in the breeding population while decreasing the frequency of the unfavorable alleles (Hallauer, 1985; Hallauer and Miranda, 1988; Mhike et al., 2010). Our result is in agreement with Bernardo and Mayor (2009), who reported an increase in frequency of favorable marker alleles for grain yield, grain moisture, plant integrity, and stay green in a DH mapping population improved using MARS. Edwards and Johnson (1994), also reported an increase in the frequency of favorable alleles in an F_2_ population of sweet corn improved using MARS with some marker loci fixed for the favorable alleles. The frequency of alleles with large effects should increase or decrease faster than the frequency of alleles with relatively small effects (Delaney and Bliss, 1991). The trajectory of change in allele frequency allows for the identification of favorable, neutral, or unfavorable alleles. On the other hand, Johnson (2004) found a high mean frequency at C_1_ while a third population showed no difference in mean frequency of favorable alleles between C_1_ and C_2_. Our results suggest that breeder may conduct three cycles of MARS to develop superior inbred lines for evaluation.

The mean combination of favorable alleles present in the present study reveals that all the S_1_ lines derived from the advanced cycle (C_3_), had more combinations of favorable alleles than those derived from C_0_, suggesting that a lot of recombination had taken place and the breeding scheme was effective in enhancing genetic gain in the bi-parental population. The observed increase in inbreeding coefficient with selection are in agreement with Dorak (2014), who stated that positive inbreeding coefficient values indicates heterozygote deficiency compared with Hardy–Weinberg Equilibrium expectations. The high rate of loss of heterozygosity in the MARS populations was also an indicator of the effect of selection. The frequency of heterozygotes at the marker loci shown in MARS cycles decreased as the selection progressed, which was consistent with the findings of Massman et al. (2013), who also observed loss of heterozygosity in testcrosses of B73 × Mo17 MARS population using SNP markers. The unequal percentage decrease in heterozygosity and increase in homozygosity was due to loss of some genotypes in their homozygous state. The loss of genotypes observed in this study suggests that desirable alleles were selected throughout the four selection cycles. The fact that no genotype was gained throughout the selection process in each cycle provides further evidence that the genotypic data was accurately scored and is a true representation of the genetic changes that occurred during breeding cycles. A similar result was observed by Vogel (2010) in characterization of maize populations selected for grain methionine content using SNP markers. Further research is needed on the use of haplotype signatures to identify genomic regions that have responded to selection.

## Conclusion

The MARS procedure caused desirable changes in frequency of favorable marker alleles, though no significant gain in grain yield was recorded under DS condition due to the severe fall armyworm infestation. The absence of changes in grain yield from the original to the advanced selection cycles under *Striga* infestation could arise from the fact that the selection has not been done to improve defensive traits against *Striga*. Our study demonstrated that selection for improved performance under DS did not necessarily have a negative effect on grain yield under WW conditions and also, selection of parents with tolerance to multiple stress may allow some acceptable level of resistance to the parasite even when improvements were made only for tolerance to drought. To make significant progress from selection, however, selection for multiple stress should be done simultaneously under the target stress conditions. MARS could therefore, be used to improve genetic gains for complex traits like drought and accelerate the development of new inbred lines in maize breeding programs.

## Author Contributions

MG have contributed in the genotyping and cycling by GEBV selection as well as review of the manuscript; AM contributed in the breeding conception of the work, edited, critically reviewed, and gave final approval of the version of the manuscript; SM, SA, JO, and DA also reviewed the manuscript; NU contributed in the genotypic selection of the work; SH designed and optimized the experiment; JC performed biometric analysis of the parental and F_2_ generation; and RA analyzed, interpreted the data, and wrote the manuscript.

## Conflict of Interest Statement

The authors declare that the research was conducted in the absence of any commercial or financial relationships that could be construed as a potential conflict of interest.

## Footnotes

1 http://www.kbioscience.co.uk/
