# Supplementary material for: Genetic Gains in Grain Yield of a Maize Population Improved through Marker Assisted Recurrent Selection under Stress and Non-stress Conditions in West Africa
Source: Front Plant Sci. 2017 May 22;8:841. doi: 10.3389/fpls.2017.00841 (PMC5438988; doi:10.3389/fpls.2017.00841)
Supplement: Supplementary file 1 [file Data_Sheet_1.DOC]

Supplementary Material

**Genetic Gain in Grain Yield of a Maize Population Improved through Marker Assisted Recurrent Selection under Stress and Non-stress Conditions in West Africa**

**Rekiya Abdulmalik O^1,2^, Abebe Menkir^1*^, Melaku Gedil^1*^**

***Correspondence:**

Melaku Gedil

m.gedil@cgiar.org

Abebe Menkir

a.menkir@cgiar.org

**1. Supplementary Data: Marker data showing marker names, alleles and their positions.**

| **S/N** | **SNP ID** | **ALLELE** | **CHROMOSOME** | **PHYSICAL MAP POSITION (bp)** |
| --- | --- | --- | --- | --- |
| **1** | csu1171.2 | A/G | 1 | 14578606 |
| **2** | kip1.3 | C/T | 1 | 255577993 |
| **3** | PHM12323.17 | C/G | 1 | 53357797 |
| **4** | PHM14475.7 | C/T | 1 | 256245118 |
| **5** | PHM174.13 | A/G | 1 | 294904503 |
| **6** | PHM1932.51 | A/G | 1 | 118875639 |
| **7** | PHM3463.18 | A/G | 1 | 106204545 |
| **8** | PHM4053.15 | A/G | 1 | 176705478 |
| **9** | PHM4997.11 | C/A | 1 | 6230096 |
| **10** | PHM5480.17 | A/G | 1 | 204199508 |
| **11** | PHM6043.19 | A/G | 1 | 216927159 |
| **12** | PZA00175.2 | A/T | 1 | 8510027 |
| **13** | PZA00181.2 | A/C | 1 | 8366411 |
| **14** | PZA00343.31 | A/G | 1 | 294646235 |
| **15** | PZA00664.3 | A/G | 1 | 227542649 |
| **16** | PZA01019.1 | C/G | 1 | 208143724 |
| **17** | PZA01216.1 | A/G | 1 | 203035464 |
| **18** | PZA01254.2 | A/G | 1 | 106204446 |
| **19** | PZA01497.1 | A/G | 1 | 14578834 |
| **20** | PZA01588.1 | A/G | 1 | 258495441 |
| **21** | PZA01866.1 | A/C | 1 | 51576563 |
| **22** | PZA01921.19 | A/G | 1 | 261313982 |
| **23** | PZA01921.20 | A/G | 1 | 261313627 |
| **24** | PZA02044.1 | A/G | 1 | 290807362 |
| **25** | PZA02094.9 | A/T | 1 | 15725672 |
| **26** | PZA02117.1 | A/G | 1 | 223466480 |
| **27** | PZA02186.1 | A/C | 1 | 227896382 |
| **28** | PZA02698.3 | A/G | 1 | 272318990 |
| **29** | PZA02763.1 | T/C | 1 | 101421538 |
| **30** | PZA03578.1 | A/G | 1 | 252219631 |
| **31** | PZB00063.1 | A/C | 1 | 268373631 |
| **32** | PZB00872.3 | A/G | 1 | 46255059 |
| **33** | PHM12979.9 | A/G | 2 | 9037148 |
| **34** | PHM13440.13 | A/G | 2 | 2527344 |
| **35** | PHM13648.11 | C/T | 2 | 144527713 |
| **36** | PHM3334.4 | G/A | 2 | 5053894 |
| **37** | PHM3334.6 | A/G | 2 | 5053798 |
| **38** | PHM3626.3 | A/G | 2 | 125642617 |
| **39** | PHM4780.38 | A/G | 2 | 31494364 |
| **40** | PHM4951.8 | A/G | 2 | 1174438 |
| **41** | PHM5822.15 | A/T | 2 | 10429405 |
| **42** | PHM7953.11 | A/G | 2 | 195938696 |
| **43** | PZA00029.17 | A/C | 2 | 144528184 |
| **44** | PZA00613.22 | C/T | 2 | 4180789 |
| **45** | PZA00620.3 | A/T | 2 | 10429405 |
| **46** | PZA00755.2 | A/G | 2 | - |
| **47** | PZA00804.1 | A/G | 2 | 212078562 |
| **48** | PZA01280.2 | A/G | 2 | 149428645 |
| **49** | PZA02496.1 | A/C | 2 | 40814083 |
| **50** | PZA02626.1 | C/G | 2 | 144527921 |
| **51** | PZA03644.1 | A/G | 2 | 170899849 |
| **52** | PHM12859.7 | C/T | 3 | 5479991 |
| **53** | PHM13742.5 | A/G | 3 | 213606180 |
| **54** | PHM15449.10 | A/G | 3 | 125077922 |
| **55** | PHM2919.23 | A/C | 3 | 199886409 |
| **56** | PHM3352.19 | T/A | 3 | 208711808 |
| **57** | PZA00088.3 | C/G | 3 | 228614270 |
| **58** | PZA00109.4 | T/A | 3 | 82173052 |
| **59** | PZA00219.7 | A/G | 3 | 219309085 |
| **60** | PZA00279.2 | A/G | 3 | 52804070 |
| **61** | PZA00413.18 | A/G | 3 |  |
| **62** | PZA00413.20 | A/C | 3 | 125192432 |
| **63** | PZA00538.15 | A/G | 3 | 206889707 |
| **64** | PZA00667.2 | A/G | 3 | 161516227 |
| **65** | PZA00707.9 | A/T | 3 | 110715954 |
| **66** | PZA00827.1 | A/C | 3 | 155566732 |
| **67** | PZA00920.1 | A/G | 3 | 142821031 |
| **68** | PZA01154.1 | A/G | 3 | 216028207 |
| **69** | PZA01396.1 | A/G | 3 | 164833462 |
| **70** | PZA03032.19 | C/G | 3 | 171364540 |
| **71** | PZA03154.4 | C/T | 3 | 202364110 |
| **72** | PZA03458.1 | A/G | 3 | 203318583 |
| **73** | PZA03733.1 | A/G | 3 | 180532218 |
| **74** | PZB02179.1 | A/G | 3 | 157640380 |
| **75** | bt2.4 | A/G | 4 | 66288635 |
| **76** | bt2.7 | A/G | 4 | 66290994 |
| **77** | PHM14618.11 | A/C | 4 | 180315663 |
| **78** | PHM15427.11 | A/G | 4 | 33862684 |
| **79** | PHM18386.29 | G/A | 4 | 240105203 |
| **80** | PHM2438.28 | A/G | 4 | 3548007 |
| **81** | PHM259.11 | G/A | 4 | 14326091 |
| **82** | PHM3587.6 | G/A | 4 | 59448994 |
| **83** | PHM3963.33 | G/C | 4 | 5459125 |
| **84** | PHM4310.112 | A/C | 4 | 244087971 |
| **85** | PHM4901.9 | A/G | 4 | 26467663 |
| **86** | PHM4905.6 | A/T | 4 | - |
| **87** | PZA00399.11 | G/A | 4 | 229644826 |
| **88** | PZA00636.7 | A/G | 4 | 226884613 |
| **89** | PZA00941.2 | C/G | 4 | 185562016 |
| **90** | PZA01332.2 | C/T | 4 | 212893509 |
| **91** | PZA01751.2 | A/T | 4 | 37185517 |
| **92** | PZA01905.12 | A/G | 4 | 244087450 |
| **93** | PZA02289.2 | C/T | 4 | 180316143 |
| **94** | PZA02479.1 | A/G | 4 | 218367682 |
| **95** | PZA02585.2 | A/T | 4 | 239453847 |
| **96** | PZA03116.1 | A/G | 4 | 166002318 |
| **97** | PZA03270.2 | A/G | 4 | 76122802 |
| **98** | PZA03597.1 | A/G | 4 | 74239902 |
| **99** | PZB01461.1 | A/G | 4 | 194092995 |
| **100** | PHM13696.11 | G/A | 5 | 175367080 |
| **101** | PHM13942.7 | G/A | 5 | 21461779 |
| **102** | PHM13942.8 | A/G | 5 | 21461728 |
| **103** | PHM13942.9 | A/G | 5 | 21461663 |
| **104** | PHM16854.3 | C/G | 5 | 34587029 |
| **105** | PHM1899.157 | A/G | 5 | 179060561 |
| **106** | PHM2348.66 | A/G | 5 | 87895872 |
| **107** | PHM2769.43 | A/G | 5 | 57831576 |
| **108** | PHM3137.17 | A/G | 5 | 7487050 |
| **109** | PHM4647.8 | C/G | 5 | 32599447 |
| **110** | PHM5484.22 | A/C | 5 | 21449633 |
| **111** | PHM563.9 | A/G | 5 | 204993639 |
| **112** | PHM6386.11 | A/G | 5 | 190026183 |
| **113** | PHM7908.20 | A/C | 5 | 191075278 |
| **114** | PHM7908.25 | A/C | 5 | 191075472 |
| **115** | PZA00148.3 | A/C | 5 | 164230168 |
| **116** | PZA00222.7 | A/G | 5 | 58576757 |
| **117** | PZA00300.14 | A/G | 5 | 171688876 |
| **118** | PZA00352.23 | A/G | 5 | 191075557 |
| **119** | PZA00980.1 | A/G | 5 | 203778507 |
| **120** | PZA01050.1 | A/G | 5 | 63415425 |
| **121** | PZA01284.6 | A/T | 5 | 11990631 |
| **122** | PZA01304.1 | A/G | 5 | 178584058 |
| **123** | PZA01327.1 | A/G | 5 | 15056528 |
| **124** | PZA01349.2 | A/G | 5 | 69319346 |
| **125** | PZA01371.1 | A/G | 5 | 8220010 |
| **126** | PZA01438.1 | A/T | 5 | 2690742 |
| **127** | PZA01608.1 | C/T | 5 | 158599491 |
| **128** | PZA02015.11 | C/T | 5 | 207464707 |
| **129** | PZA02029.21 | C/T | 5 | 7956551 |
| **130** | PZA02164.16 | A/G | 5 | 112179855 |
| **131** | PZA02525.1 | G/T | 5 | 69392105 |
| **132** | PZA02653.12 | C/T | 5 | 5917587 |
| **133** | PZA02676.2 | C/T | 5 | 60072336 |
| **134** | PZA02769.1 | A/T | 5 | 215505432 |
| **135** | PZA02981.2 | A/T | 5 | 166468431 |
| **136** | PZA03092.7 | C/T | 5 | 11992705 |
| **137** | PZA03340.2 | G/C | 5 | 20201009 |
| **138** | PZA03452.6 | C/T | 5 | 182823983 |
| **139** | PZB01112.1 | C/G | 5 | 68419652 |
| **140** | PZB01115.3 | C/T | 5 | 60964334 |
| **141** | PHM1190.3 | A/G | 6 | 120234344 |
| **142** | PHM15251.3 | A/G | 6 | 153123339 |
| **143** | PHM15251.5 | A/T | 6 | 153123234 |
| **144** | PHM15961.13 | C/G | 6 | 9561803 |
| **145** | PHM4503.25 | A/C | 6 | 161137975 |
| **146** | PHM5529.4 | C/A | 6 | 167120845 |
| **147** | PHM5794.13 | A/G | 6 | 156740198 |
| **148** | PZA00266.7 | C/T | 6 | 161570136 |
| **149** | PZA00440.15 | A/G | 6 | 22403926 |
| **150** | PZA01072.1 | A/C | 6 | 137479485 |
| **151** | PZA01468.1 | A/G | 6 | 161663671 |
| **152** | PZA01591.1 | A/G | 6 | 125113941 |
| **153** | PZA01672.1 | A/G | 6 | 154495634 |
| **154** | PZA01729.1 | A/G | 6 | 123998706 |
| **155** | PZA02148.1 | A/G | 6 | 143875945 |
| **156** | PZA02815.25 | A/G | 6 | 167883450 |
| **157** | PZA03047.12 | A/G | 6 | 20837504 |
| **158** | PZB01658.1 | A/T | 6 | 102953833 |
| **159** | PHM12830.14 | A/G | 7 | 71717792 |
| **160** | PHM1912.20 | G/A | 7 | 155970323 |
| **161** | PHM1912.23 | G/C | 7 | 155970264 |
| **162** | PHM4135.15 | C/G | 7 | 6437899 |
| **163** | PHM4353.31 | A/C | 7 | 36392983 |
| **164** | PHM7898.10 | A/C | 7 | 161993743 |
| **165** | PHM9162.135 | A/G | 7 | 137834663 |
| **166** | PZA00111.10 | C/T | 7 | 133825874 |
| **167** | PZA00418.2 | A/G | 7 | 71717601 |
| **168** | PZA01946.7 | A/C | 7 | 123601837 |
| **169** | PZA02260.2 | C/G | 7 | 153012827 |
| **170** | PZA02854.13 | A/G | 7 | 137834376 |
| **171** | PZA03166.1 | A/G | 7 | 137632654 |
| **172** | PZA03363.1 | A/G | 7 | 49538583 |
| **173** | PZA03645.1 | C/T | 7 | 73892322 |
| **174** | PHM10525.11 | A/G | 8 | 124752481 |
| **175** | PHM10525.9 | A/C | 8 | 124752624 |
| **176** | PHM15744.10 | A/G | 8 | 134813437 |
| **177** | PHM4134.8 | C/G | 8 | 105795742 |
| **178** | PHM4786.9 | A/G | 8 | 164795880 |
| **179** | PHM523.21 | A/G | 8 | 136015581 |
| **180** | PHM934.19 | A/G | 8 | 116786833 |
| **181** | PZA00739.1 | A/G | 8 | 105796017 |
| **182** | PZA00760.1 | C/G | 8 | 171099906 |
| **183** | PZA00770.1 | C/G | 8 | 134140609 |
| **184** | PZA00793.2 | A/C | 8 | 64421988 |
| **185** | PZA01038.1 | A/G | 8 | 123599285 |
| **186** | PZA02011.1 | A/G | 8 | 140212456 |
| **187** | PZA02174.2 | G/T | 8 | 4101256 |
| **188** | PZA02388.1 | A/G | 8 | 169137 |
| **189** | PZA03637.1 | A/G | 8 | 118439775 |
| **190** | PZA03638.1 | A/T | 8 | 118438296 |
| **191** | PZA03650.1 | A/T | 8 | 146342067 |
| **192** | PZA03651.1 | A/C | 8 | 146342784 |
| **193** | PZB01454.1 | G/A | 8 | 145713853 |
| **194** | PHM1766.1 | C/G | 9 | 136401627 |
| **195** | PHM4942.12 | A/G | 9 | 3166446 |
| **196** | PZA00225.8 | A/G | 9 | 104537847 |
| **197** | PZA01062.1 | A/C | 9 | 88057320 |
| **198** | PZA01096.1 | G/T | 9 | 133450713 |
| **199** | PZA01715.1 | A/G | 9 | 142948545 |
| **200** | PZA01715.2 | C/T | 9 | 142948449 |
| **201** | PZA02235.14 | C/T | 9 | 132119630 |
| **202** | PZA03670.1 | G/T | 9 | 130886940 |
| **203** | PZB01899.1 | A/G | 9 | 98502843 |
| **204** | sh1.12 | A/G | 9 | 11340882 |
| **205** | PHM13687.14 | A/G | 10 | 117796822 |
| **206** | PHM1576.25 | A/G | 10 | 124203168 |
| **207** | PHM1752.36 | A/G | 10 | 9746552 |
| **208** | PHM2770.19 | A/C | 10 | 72565410 |
| **209** | PHM2828.83 | C/T | 10 | 6121374 |
| **210** | PHM4066.11 | A/G | 10 | 41187565 |
| **211** | PZA00007.1 | A/C | 10 | 144172316 |
| **212** | PZA00048.1 | C/T | 10 | 98582149 |
| **213** | PZA00463.3 | A/G | 10 | 13546326 |
| **214** | PZA01073.1 | A/G | 10 | 144549995 |
| **215** | PZA01089.1 | C/T | 10 | 117372550 |
| **216** | PZA01241.2 | A/C | 10 | 130452676 |
| **217** | PZA01292.1 | C/T | 10 | 109633741 |
| **218** | PZA02049.1 | C/G | 10 | 144025574 |
| **219** | PZA02398.2 | C/T | 10 | 99471436 |
| **220** | PZA02470.2 | C/T | 10 | 18714209 |
| **221** | PZA02853.11 | C/G | 10 | 25966022 |
| **222** | PZA02961.6 | A/T | 10 | 16264897 |
| **223** | PZA02969.9 | C/T | 10 | 143676084 |
| **224** | PZA03196.1 | A/G | 10 | 124005403 |
| **225** | PZA03603.1 | A/T | 10 | 141832715 |
| **226** | PZA03605.1 | A/G | 10 | 141830532 |
| **227** | PZA03606.1 | A/T | 10 | 141829598 |
| **228** | PZA03607.1 | C/T | 10 | 141828531 |
| **229** | PZA03713.1 | A/T | 10 | 121489957 |
| **230** | PZB01111.8 | A/C | 10 | 134034407 |
| **231** | PZA00311.5 | G/A | Unknown | unknown |
| **232** | PZA02167.2 | A/G | 10 | unknown |
| **233** | PZA02397.1 | C/G | 9 | unknown |
